# Comparable worth of life for all? Conducting and disseminating health economic evaluations for refugees in Germany

**DOI:** 10.1186/s12992-022-00845-1

**Published:** 2022-05-12

**Authors:** Louise Biddle, Katharina Wahedi, Kayvan Bozorgmehr

**Affiliations:** 1 Section for Health Equity Studies and Migration, Department of General Practice and Health Services Research, University Hospital Heidelberg, Heidelberg, Germany; 2 Department of Population Medicine and Health Services Research, School of Public Health, Bielefeld University, Bielefeld, Germany

**Keywords:** Economic evaluation, Evidence dissemination, Refugees, Health policy, Screening policies, Equity

## Abstract

Comparative health economic evaluation is based on premise of being able to compare the worth of a year of life lived in full quality across different patients, population groups, settings and interventions. Given the rising numbers of forcibly displaced people, the nexus of economics, migration and health has emerged as a central theme in recent conceptual and empirical approaches. However, some of the assumptions made in conventional economic approaches do not hold true in the decision-making context of migration and the health of forcibly displaced populations. Using the experience of conducting and disseminating economic analyses to support decision-making on health screening policies for refugees in Germany, we show that in particular the assumptions of individual utility with no positive externalities, equity-blind utilitarian ethical stances and stable budgets are challenged. The further development of methods to address these challenges are required to support decision-makers in this contentious and politically fraught context and continue to make choices and decisions transparent.

## Background

Comparative health economic evaluation is based on the premise of being able to compare the worth of a year of life lived in full quality across different patients, population groups, settings and interventions [1]. The introduction of the quality-adjusted life-year (QALY) as the yardstick for measuring the economic worth of interventions has transformed modern health economics and allowed for the allocation of limited health budgets across a range of diverse promotive, preventative and curative health interventions and policies [2].

In countries which work with explicit cost-effectiveness thresholds, however, it has been shown that equal worth for a QALY does not always hold true. In the UK, for example, higher cost-effectiveness thresholds have been applied for rare diseases, cancer medication and end-of-life care [3], with regulators recommending treatments that do not necessarily represent the most cost-effective distribution of resources, but that are deemed particularly desirable from a social and moral standpoint. While such decisions–linking resource allocation with normative judgements–are contentious, the consistent use of explicit measures of cost and effect allows for a transparent deviation from previously accepted norms and an open debate about the necessity and ethical considerations of such decisions. In other contexts, these decisions are still made, but their (normative) reasoning is less transparent.

The number of refugees and asylum seekers is rising globally, and more than 1 % of the world’s population, i.e. 1 in 95 people, is now forcibly displaced [4]. To date, very few studies have considered the cost-effectiveness of health care interventions in the context of refugees and forcibly displaced populations. Economic evaluation of actions (or non-action) related to health care and health needs of these populations will be increasingly relevant to guide decision-making in receiving countries. Furthermore, economic arguments are at the core of migration and immigration policy debates, but economic evidence to back decisions or policy choices is scarce [5]. This has become even more relevant in the light of the detrimental consequences of the COVID-19 pandemic for migrants and forcibly displaced populations [6]. A research agenda has been proposed to address this gap in evidence and foster the methodological, conceptual and empirical advances at the nexus of economic, migration, and health research [5].

In this commentary, we contribute to this research agenda by reporting and reflecting upon our experience of conducting and disseminating economic evaluation studies for refugee populations. Germany, as a decision-making context in Europe which does not necessarily require cost-effectiveness analyses to support the introduction of new health technologies and interventions, commonly uses the “efficiency frontier” methodology rather than a cost-effectiveness threshold [7]. This means that the use of economic evaluations in healthcare decision-making is still in its infancy in Germany. Drawing on three studies conducted in Germany, the examples question the rationality behind some of these policies and highlight the *implicit* assumptions made by decision-makers when choosing for or against a particular intervention in a particularly contested political space: the health care of refugees. These insights demonstrate the importance of conducting meaningful economic evaluations, especially in national contexts where economic evaluations are not used systematically. What is more, they also illuminate some of the methodological and conceptual shortcomings of current economic evaluations in the context of forced displacement.

## Conducting and disseminating economic evaluation studies for refugee populations: experiences from three studies in Germany

The three studies considered here looked specifically at health screening policies for newly arriving refugees. Many countries in Europe and elsewhere conduct health screening programmes for refugees, but the range of tests provided vary markedly between and within countries. The first economic analysis [8] concerned itself with the issue of screening for mental health issues. While mental health screening for refugees is popular in the United States of America, its application in countries of Europe and the Middle East and North Africa (MENA) region is scarce [9]. It is not routine practice in Germany, but has been suggested as an intervention to improve access to essential mental health services for refugees, who are known to have a high burden of disease and multiple barriers in accessing care. However, no screening programme existed to evaluate (cost-) effectiveness in this context. The authors therefore evaluated a screening programme for depression using an economic modelling approach and uniting data from numerous sources. Results showed that screening for depression can certainly be cost-effective, with sensitivity analyses showing that this result remained stable despite the uncertainty of model and parameter assumptions made.

However, not all screening interventions are sensible. In Germany, the range of mandatory screening interventions for refugees differs by federal state. A second economic evaluation [10] set out to compare these policies, and found that many states employ screenings which are not evidence-based, such as indiscriminate stool examinations or mandatory screening for HIV. Conducting a costing study of these programmes, the authors demonstrated that excessive amounts of money were spent on finding rare diseases (e.g. >€80.000 for a case of Shigella) and that €3.1 million per year could be saved and re-invested if these screening interventions were stalled.

But in some cases, the question is not whether or not to screen, but who to screen. Refugees are a highly heterogeneous population, and indiscriminate screening programmes are not necessarily the most sensible approach. This is the case for tuberculosis (TB), where the risk of infection depends (amongst other aspects) on several risk factors including socio-economic status, country of origin, and living conditions. A third economic evaluation [11] aimed to understand the cost-effectiveness of the current German practice of indiscriminate TB screening in comparison to a targeted screening approach, in this case using TB incidence in the country of origin to determine a screening threshold. The authors found that screening only individuals from countries with a high incidence of TB, up to 50/100,000 inhabitants, came at relatively moderate costs at around 15,000€ per case found. However, widening the population group from any screening threshold to an indiscriminate screening came at a large cost of €110.000 per additional case found. This is because an incredibly large number of people at very low risk of disease have to be screened to find just a few cases. This is problematic especially in times of high in-migration as was the case during the years 2015 and 2016. The authors conclude that indiscriminate TB screening does not represent an efficient use of resources, especially as other, more (cost-)effective public health interventions, including both preventive strategies and other screening approaches, may exist for TB. This evidence can inform policy and practice not only in Germany, but also in other countries receiving refugees: recent studies have shown that globally, receiving countries use a wide variety of screening approaches, including indiscriminate screening (e.g. Kuwait, Israel, Belgium, Sweden), eligibility based on countries of origin with the highest number of refugees (e.g. Qatar, Oman, UAE) or eligibility based on varying incidence thresholds (e.g. Netherlands, UK, USA) [12, 13].

The three studies presented above demonstrate considerable benefits in terms of translatability for policy decisions: they uncover areas where further research is needed, critically question whether some common practices represent an efficient use of available resources and can guide the design of evidence-based policies. However, so far the implementation of recommendations has been limited. The reactions to these results which we have encountered when disseminating these finding to politicians and decision-makers in Germany illustrate that the assumptions made in health economic research stand in a stark contrast to the realities of the political context. In particular, two quite contradictory responses to our research demonstrate the difficulty of putting a neutral value on health interventions for refugees.

The first response, commonly heard in response to the evaluation of mental health screening, was that while the intervention proved to be cost-effective, scaling up such an intervention for the entire population of refugees would simply be too expensive. Incremental cost-effectiveness ratios (ICERs) are commonly used in economic evaluations as economists are traditionally concerned with analyses at the margin: what additional benefits can we reap with additional investments? Furthermore, ICERs allow us to compare the value of different interventions across diseases and population groups, under the assumption that a year lived in full quality is universal for all [1]. But the ICER does not give us a sense for the total costs of an intervention. However, this is what decision-makers in the context of refugee health are usually concerned with, for two main reasons: First, because of concerns that the number of refugees may vary greatly over time and providing the intervention to everyone may explode available budgets. Secondly, out of the fear of political repercussions from providing “more” or “better” healthcare to asylum seekers than for the resident population. This argument, in fact, shows a hesitancy towards the principle of vertical equity, i.e. allocation of resources towards those with highest needs, and highlights an area in which efficiency concerns intersect with equity considerations beyond conventional equity-efficiency trade-offs [14].

Paradoxically, however, we also encounter the exact opposite argument, usually in the realm of infectious diseases. That is, that some screening interventions, while expensive or not cost-effective, are simply “worth it” from the perspective of decision-makers. These concerns over health security cause large amounts of money to be spent on screening for infectious diseases [15]. In some cases, the rationale given for such large expenditures is to prevent large-scale outbreaks in the large and often crowded refugee reception centres. Interestingly, however, the cramped living conditions themselves, that are associated with higher health expenditures [16] and put the refugees at higher risk in the first place, e.g. during the COVID-19 pandemic [6], are rarely called into question. Other decision-makers are explicit about their fears that infectious diseases may spread to the German population. While it may be politically unpalatable to be seen as providing “more” or “better” healthcare to refugees, at the same time a perception of “protecting” the German population is viewed favourably (whatever it may cost).

The responses and discussion around these economic analyses provide an insightful illustration of the decision-making context of refugee health. In particular, we see some of the assumptions underpinning conventional economic evaluation approaches being called into question. Firstly, economic evaluation fundamentally uses a utilitarian approach, seeking to maximise the utility of a constrained budget across a population. In the context of refugee health, however, the question becomes: utility for whom? In empirical research, utility is frequently translated into changes in utility decrements for particular illnesses or conditions on an individual level. However, these approaches do not capture the positive externalities which may arise from, for example, infectious disease control for other members of the community.

Furthermore, conventional health economic approaches are blind to the consideration of equity [17, 18]. Utilitarian approaches allocate resources to individuals and groups which will reap the most health benefits, irrespective of whether these are individuals which already enjoy a high standard of health or whether they are very ill. However, the decision-making context is fundamentally concerned with aspects of equity (implicitly or explicitly), and this becomes particularly evident in the refugee context: decisions on who should have access to limited resources are influenced by ethical, political and social concerns; which in turn determine whether health equity is defined as a goal of resource distribution. Deriving empirical equity weights and adjustments [19] could help to make the implicit equity considerations and related decision-making processes more transparent.

Finally, the common practice of working with willingness-to-pay thresholds is based on a *stable* budget and a *stable* population, neither of which is given in the refugee health context. In Germany, health financing for refugees is based on a series of devolved budgets at several administrative levels, which have to be periodically renegotiated in a political process. Separate administrative bodies responsible for the reception and accommodation of refugees, on the one hand, and their health and social care on the other, means that budgets are highly fragmented [20]. Comparing the value of interventions across the population of refugees in Germany, and even with the German population, then, becomes highly problematic. In addition to economic evaluation studies, the financial stability of the health system should be considered and improved in order to aid economic efficiency in this context.

These issues are increasingly important in a world grappling with the effects of the Covid-19 pandemic [5]. This pandemic has made evident the deep health inequities which exist between different groups in our societies and has sparked renewed interest in the allocation of limited resources. In the context of refugees and asylum seekers, policies of “collective quarantine” [21], where refugee reception centres are shut off from the outside world without the possibility of maintaining social distance within the facilities, have made apparent the priorities of decision-makers and public health authorities primarily concerned with the health of their voter base rather than individuals at highest risk. Going forward, health economic evaluation can fundamentally contribute to the continued transparency of decision-making processes, allowing researchers and the public to scrutinise the decisions made and work towards policy solutions that are based on economic evidence and public values.

While the research outlined above was conducted in Germany, the issues raised are applicable beyond Germany and the European context. Given the high density of refugees and the complexity of financing arrangements in MENA countries, economic analyses are particularly salient in this setting [22–24]. The protracted nature of conflicts in the Middle East and West Africa, as well as the continued emergence of new conflicts such as the most recent war in the Ukraine, calls for greater scrutiny of the cost-effectiveness of health care delivery in all countries receiving refugees, as well as the values underpinning decisions made not just by national governments but also by donor agencies. To be valuable in the refugee context and, ultimately, the broader migration context [5], methodological challenges outlined here urgently need to be overcome. Indeed, the complexities brought forward by large-scale migrations can act as a catalyst to push forward the science of health economics [25].

## Conclusions

The case of conducting economic evaluation to assess the cost-effectiveness of screening interventions for refugees in Germany shows that many of the assumptions made in conventional economic evaluation approaches are not sufficient. Specifically, the further development of methods to take account of the positive externalities of health interventions, apply equity weights and assess the financial stability of health systems are needed. These ideas are not new, but their emergence in the context of forced migration makes evident that these methodological developments are not simply academic fancies but are required in practical policy decisions to ensure the health of potentially marginalised populations. Furthermore, this case study demonstrates the fundamental value of health economic evaluation in making transparent the underlying ethical and moral considerations made by decision-makers when implementing health policies, which can include racist, discriminatory and restrictive intentions towards particular groups. Sound health economic evaluations in diverse country contexts are sorely needed to continue this important work.

## Data Availability

Not applicable.

## Abbreviations

QALY: Quality-adjusted life-year
TB: Tuberculosis
ICER: Incremental cost-effectiveness ratio
